# Effect of Stopping Cotrimoxazole Preventive Therapy on Microbial Translocation and Inflammatory Markers Among Human Immunodeficiency Virus–Infected Ugandan Adults on Antiretroviral Therapy: The COSTOP Trial Immunology Substudy

**DOI:** 10.1093/infdis/jiz494

**Published:** 2019-10-23

**Authors:** Jacqueline Kyosiimire-Lugemwa, Zacchaeus Anywaine, Andrew Abaasa, Jonathan Levin, Ben Gombe, Kenneth Musinguzi, Pontiano Kaleebu, Heiner Grosskurth, Paula Munderi, Pietro Pala

**Affiliations:** 1 Medical Research Council/Uganda Virus Research Institute and London School of Hygiene and Tropical Medicine Uganda Research Unit, Entebbe, Uganda; 2 School of Public Health, University of the Witwatersrand, Johannesburg, South Africa; 3 Department of Infectious Disease Epidemiology, London School of Hygiene and Tropical Medicine, London, United Kingdom; 4 International Association of Providers of AIDS Care, Washington, District of Columbia, USA

**Keywords:** HIV-1, cotrimoxazole preventive therapy, inflammation, microbial translocation, immune activation, ART

## Abstract

**Background:**

Cotrimoxazole preventive therapy (CPT) in human immunodeficiency virus (HIV) infection is a World Health Organization–recommended standard of care in resource-limited settings, but the mechanism of CPT’s beneficial effects is unclear. The COSTOP trial (ISRCTN44723643) evaluated the noninferiority of discontinuing CPT in stabilized patients on antiretroviral therapy. The COSTOP immunology substudy was conducted on a subset of COSTOP participants randomized to continue CPT (n = 86) or discontinue CPT (placebo, n = 86) as daily treatment for 1 year.

**Methods:**

We evaluated whether CPT reduces microbial translocation, indicated by the presence of bacterial lipopolysaccharide (LPS) and LPS control factors such as soluble CD14 (sCD14) and endotoxin core antibody (EndoCAb immunoglobulin M [IgM]) in plasma. Intestinal barrier damage as indicated by plasma intestinal fatty acid binding protein (IFABP), T-cell activation, and the inflammatory markers C-reactive protein (CRP), interleukin 6 (IL-6), and tumor necrosis factor α (TNF-α) were also evaluated.

**Results:**

We found no significant change in markers of microbial translocation (LPS, IFABP, sCD14, and T-cell activation), with decreased EndoCAb IgM. There was significant increase in inflammation markers (CRP and IL-6) after stopping CPT compared to those who continued CPT.

**Conclusions:**

These results add to the evidence of immunological benefits of CPT among HIV-infected populations in resource-limited settings. However, no evidence of reducing microbial translocation was observed.

## Teaser

Cotrimoxazole preventive therapy in antiretroviral therapy–treated adults with HIV infection reduces inflammatory markers but has no effect on markers of microbial translocation, intestinal barrier integrity, or T-cell and monocyte activation.

**(See the Editorial Commentary by Bourke and Prendergast, on pages 347–50.)**

Cotrimoxazole preventive therapy (CPT) reduces morbidity and mortality in people with untreated human immunodeficiency virus (HIV) infection [1, 2] and in those on antiretroviral therapy (ART) [3, 4]. The mechanism underlying this benefit of CPT remains unclear. Cotrimoxazole is a combination of sulfamethoxazole and trimethoprim with a broad spectrum of activity against bacteria as well as activity against *Plasmodium falciparum* (malaria) due to the sulfamethoxazole component [5]. CPT might therefore act by causing a reduction in intercurrent infections. However, the benefit of CPT has been demonstrated in the presence of a high prevalence of antimicrobial resistance to CPT, and CPT has furthermore been associated with a benefit in infections caused by pathogens against which CPT is not known to have biological activity such as *Mycobacterium tuberculosis*, some other bacteria, and helminths [6–9]. One possible mechanism could be a nonspecific lowering of the bacterial load in the gastrointestinal tract or limiting damage to the mucosal epithelium, resulting in lower concentrations of microbial products crossing the intestinal mucosal barrier and consequent effect on reducing immune activation and inflammation.

HIV type 1 infection depletes the gut-associated CD4 T cells and damages the ability of the gut mucosal barrier to exclude microbial products present in the lumen from entering the bloodstream [10]. Once in the bloodstream, lipopolysaccharide (LPS) derived from gram-negative bacteria endotoxin and fungal products is captured by the LPS binding protein, which carries it to the CD14 receptor [11] on the membrane of monocytes, neutrophils, epithelial cells, and dendritic cells. CD14 brings LPS into contact with Toll-like receptor 4 (TLR4), stimulating activation of the innate immune system either directly or in synergy with TREM-1 (triggering receptor expressed on myeloid cells 1) [12]. Thus, bacterial LPS contributes to systemic inflammation, raising levels of circulating C-reactive protein (CRP), interleukin 6 (IL-6), tumor necrosis factor α (TNF-α), and other cytokine/chemokines. Alternative mechanisms used to control LPS include host generation of natural antibodies against the endotoxin core (EndoCAb). These antibodies form complexes with circulating LPS and can become depleted from the plasma in the process [13]. The imbalance between LPS and endotoxin core antibodies may contribute to the general immune activation of T cells [14] and the inflammation that characterizes chronic HIV infection and which is associated with loss of CD4 cells and progression to AIDS [10].

In a cohort study of HIV-uninfected subjects from Entebbe, Uganda, baseline levels of inflammatory cytokines were found to be more elevated than in a cohort based in Lausanne, presumably because of higher exposure to environmental infections [15]. We hypothesized that in epidemiological contexts such as that prevailing in Uganda, CPT reduces levels of unspecified bacterial infections, including levels of intestinal bacteria, and therefore the risk of intestinal microbial translocation. We evaluated whether CPT discontinuation is associated with increased microbial translocation, immune activation, and elevated levels of inflammatory markers.

## MATERIALS AND METHODS

### Study Design

The COSTOP trial (Safety of discontinuing cotrimoxaxole prophylaxis among human immunodeficiency virus (HIV) infected adults in Uganda: a randomised controlled trial, ISRCTN44723643) was a randomized, double-blind, placebo-controlled noninferiority trial among 2180 HIV-infected and stabilized patients on ART with CD4 counts ≥250 cells/μL [16]. Findings of this trial showed that continued CPT significantly reduced the risk of severe bacterial infections and protected against malaria, whereas discontinuing CPT reduced hematological adverse events [17]. Within the COSTOP trial, the COSTOP immunology substudy was conducted on a subset of 172 COSTOP study participants, independently randomized and recruited toward the end of the trial’s enrollment phase. Like all other volunteers in the COSTOP trial, participants for the immunology study were randomly assigned to 1 of 2 study arms: 1 arm (n = 86) continued with CPT, and the other arm (n = 86) discontinued CPT and was given placebo. Blood samples were collected at baseline and at 3, 6, and 12 months.

### Study Settings and Participants

The COSTOP immunology study was conducted at the Masaka Regional Referral Hospital (MRRH). Study participants were adult male and female HIV-infected Ugandans enrolled at 4 outpatient HIV care clinics located near or within MRRH. The hospital serves a periurban and rural population. All patients were stable on ART and at enrollment had CD4 counts of ≥250 cells/µL and had been taking CPT as standard of care for at least 6 months. Patients were excluded if they had CPT hypersensitivity, had an acute febrile illness, were in the first trimester of pregnancy, or had a grade 3 or 4 anemia, neutropenia, or thrombocytopenia.

Ethical approvals were obtained from the Uganda Virus Research Institute Research and Ethics Committee, the Ugandan National Council for Science and Technology, and the Uganda National Drug Authority. All participants gave written informed consent. Illiterate participants indicated their consent by thumbprint in the presence of an impartial literate witness.

### Laboratory Investigations

LPS was considered as a marker of microbial translocation; intestinal fatty acid binding protein (IFABP) as a marker of intestinal barrier damage; soluble CD14 (sCD14) as a marker of monocyte activation or response to LPS; expression of HLA-DR and CD38 as markers of CD4 and CD8 T-cell activation; and anti-endotoxin core antibodies (EndoCAb immunoglobulin M [IgM]) as a marker of control of circulating LPS. We also assessed IL-6, TNF-α, and CRP as markers of inflammation.

Ethylenediaminetetraacetic acid (EDTA) and heparin anticoagulated whole blood samples were collected at baseline and at 3, 6, and 12 months. Plasma was separated from whole blood, aliquoted, and stored at –80°C for later batch measurement of plasma markers of microbial translocation and inflammation. Sterile endotoxin-free tubes were used.

#### Microbial Translocation

LPS levels in plasma were measured with the limulus amoebocyte lysate (LAL)–based test with Endosafe portable test system (PTS) cartridges (catalog number PTS200005F; range, 0.5–0.005 EU/mL) for endotoxin microbial detection (Charles River) and read using an Endosafe PTS reader (Charles River). LPS levels were reported as endotoxin units (EU)/mL. Heparinized plasma was diluted 1:50 with LAL reagent water (LRW) to dilute any inhibitors. The diluted plasma was heated at 80°C for 10 minutes and LPS levels were measured using the Endosafe PTS endotoxin method, which included an internal control with a known endotoxin spike to correct the effect of any residual inhibitors. The negative control was LRW and the external positive control was a reference standard endotoxin (Charles River).

#### T-Cell Activation

Fresh EDTA whole blood samples (500 μL) were stained with antibodies CD3-QD655 (clone S4.1, Invitrogen), CD4-QD605 (clone S3.5, Invitrogen), CD8-PECy7 (clone 3B5, BD Biosciences), HLA-DR-FITC (clone G46-6, BD Biosciences), and CD38-APC (clone HIT2, BD Biosciences). CD14-Pacific blue (clone TÜK4) and CD19-Pacific blue (clone SJ25-C1) from Invitrogen were used to exclude monocytes and B cells. Stained cells were acquired on an 18-color LSR II flow cytometer (BD Immunocytometry Systems) and analyzed using FlowJo software. The gating strategy (Supplementary Figure 2) was to gate on lymphocytes in the scatterplot, then singlets, then live CD3^+^ and CD14^–^ and CD19^–^ cells. From the CD3^+^ cell population, CD4^+^ and CD8^+^ cell populations were selected. Activation was measured as percentage of CD38^+^HLA-DR^+^CD8^+^ or CD38^+^HLA-DR^+^CD4^+^ T cells.

#### Inflammation, sCD14, Epithelial Intestinal Damage, and Anti-endotoxin Core Antibodies

Enzyme-linked immunosorbent assays (ELISAs) were performed on stored plasma to determine the presence and levels of sCD14 (catalog number DC140; range, 250–16 000 pg/mL), IL-6 (catalog number S6050; range, 1.56–100 pg/mL), TNF-α (catalog number SSTA00C; range, 3.9–250 pg/mL), CRP (catalog number SCRP00; range, 0.78–50 ng/mL) (all from Quantikine immunoassays, R&D Systems Europe Ltd); IFABP (catalog number HK406-02; range, 47–3000 pg/mL), and EndoCAb (catalog number HK-504-IgM; range, 0.05–3.5 MMU/mL) (Hycult Biotech). Absorbance values were read using the BioTek U Quant microplate reader (catalog number 285179), and Gen5 software was used to interpret plate readings. Standard operating procedures were designed for each of these assays (according to the manufacturer’s recommendations) and validity of the results included assessing the *R*^2^ of each standard curve (>0.9) and intra-assay/interassay validation %coefficient of variation of ≤ 15.

### Statistical Methods

The sample size of 86 subjects per arm was determined based on comparing the primary outcome of LPS at 12 months between subjects who would continue CPT and subjects who would stop CPT at enrollment (ie, would take placebo). Assumptions were based on an earlier study comparing long-term nonprogressors (LTNPs) to rapid progressors, where it was found that the mean LPS levels differed between these 2 groups with a mean of 665 pg/mL in the LTNP group and a mean of 757 pg/mL in the rapid progressor group (*P* = .0001), with a pooled within-group standard deviation (SD) of 54.6 pg/mL (J. Kyosiimire-Lugemwa, unpublished data). In the main COSTOP trial, all subjects had been on CPT and ART before enrollment, and due to the randomization we assumed that the 2 arms were likely to be very similar at baseline with respect to the risk of intestinal microbial translocation. It was therefore also likely that the difference in LPS levels at 12 months after CPT cessation would be smaller than that observed in the study of LTNPs and rapid progressors. Therefore, a sample size of 76 subjects per arm would have 80% power to detect as statistically significant at the 5% level a true mean difference of 25 pg/mL between subjects who continued CPT and those who stopped CPT for 12 months, assuming a pooled within-group SD of 55 pg/mL in LPS. For purposes of comparison of LPS units between the LTNP study mentioned above and the current study, 100 pg/mL is equivalent to 1 EU/mL; thus, 25 pg/mL is equivalent to 0.25 EU/mL. With the assumption of smaller difference in LPS levels at 12 months, the sample size of 76 subjects per arm formed the sample size for the primary outcome investigated in the substudy. However, to cater for loss to follow-up of up to 10 patients, the sample size was increased to 86 patients per arm. Furthermore, this sample size would also provide adequate power for investigation of the secondary outcomes.

LPS and all ELISA data were transcribed from the reader output into Excel spreadsheets; flow cytometry standard files from DiVA were analyzed with FlowJo; and cell frequencies were tabulated into Excel spreadsheets. Data were cleaned and analyzed using Stata 14 software (StataCorp). Participant baseline sociodemographics and clinical characteristics were summarized by trial arm using frequencies and percentages for categorical variables, and mean and SD or median and interquartile range for continuous variables. The analysis was by intention to treat. We displayed the geometric mean markers by trial arm and over the follow-up time using line graphs and estimated the trend *P* value in each plot using a linear regression model controlling for the baseline value of each marker. Because the concentrations of LPS showed skewed distributions with 111 patients (59 cotrimoxazole and 52 placebo) having values below the minimum quantification threshold, a constant was added to each marker value before transformation on the natural logarithmic scale and subsequent analysis. Frequencies of activated CD4 and CD8 T cells were not transformed. We compared the means of markers between subjects in the 2 trial arms at baseline and at 3, 6, and 12 months. Linear mixed models for longitudinal data were fitted for each marker, using the values at baseline, month 3, month 6, and month 12, with random terms for subject and random slopes over time, to compare the average slope over time in the 2 trial arms. The linear mixed models were fitted, first without a time interaction term and then including a time interaction term. Relationships between markers were evaluated by Spearman rank correlation and with Bonferroni correction for multiple comparisons.

## RESULTS

Between 2012 and 2014, we enrolled 172 volunteers into the immunology substudy conducted in Masaka. Volunteers were randomized (1:1) to either continue CPT (n = 86) or to discontinue treatment (placebo, n = 86) and were followed up for 1 year (Supplementary Figure 1). The baseline characteristics of the 2 arms are summarized in Table 1. Randomization achieved a balanced baseline distribution between trial arms with regards to participants’ age, sex, sociodemographic and physical characteristics, stage of HIV infection, and current and past treatment regimens (Table 1). During the study, only 4 cases of malaria occurred (2 in each arm). Retention was good, with 162 (94.2%) volunteers returning for their final month 12 study visit (80 and 82 in the CPT and placebo arms, respectively; Supplementary Figure 1). The numbers of study participants and their samples analyzed for each biomarker (from baseline to 12 months) are shown in Supplementary Table 1.

**Table 1.** Baseline Characteristics of the Study Participants by Trial Arm (N = 172)

Variable	CPT (n = 86)	Placebo (n = 86)
CD4 stratum		
<500 cells/μL	41 (47.7)	42 (48.8)
≥500 cells/μL	45 (52.3)	44 (51.2)
CD4 count at randomization, cells/μL		
Median (IQR)	534 (418–706)	537 (432–746)
250–349	5 (5.8)	6 (7.0)
350–499	36 (41.9)	36 (41.9)
≥500	45 (52.3)	44 (51.1)
Sex		
Male	18 (20.9)	24 (27.9)
Female	68 (79.1)	62 (72.1)
Age, y		
Mean (SD)	41.4 (9.1)	42.3 (10.4)
Median (IQR)	41 (36–47)	43 (34–50)
Weight, kg, mean (SD)	57.2 (10.3)	60.3 (11.3)
BMI, kg/m^2^, mean (SD)	22.5 (3.7)	23.4 (4.4)
WHO stage		
I	9 (10.5)	5 (5.8)
II	22 (25.6)	22 (25.6)
III	51 (59.3)	54 (62.8)
IV	4 (4.6)	5 (5.8)
Educational level		
None	6 (7.0)	9 (10.5)
Primary	60 (69.8)	59 (68.6)
Secondary	20 (23.2)	16 (18.6)
University/vocational	0 (0.0)	2 (2.3)
Marital status		
Married/cohabiting	35 (40.7)	31 (36.1)
Separated/divorced	47 (54.6)	50 (58.1)
Single, never married	4 (4.7)	5 (5.8)
HIV status disclosure to partner		
Yes	43 (50.0)	42 (48.8)
No	3 (3.5)	4 (4.7)
Unspecified	40 (46.5)	40 (46.5)
Months on ART, median (IQR)	50 (25–80)	43 (17–73)
Initial ART regimen		
AZT/3TC/NVP	37 (43.0)	40 (46.5)
AZT/3TC/EFV	9 (10.5)	5 (5.8)
d4T/3TC/NVP	19 (22.1)	15 (17.4)
d4T/3TC/EFV	1 (1.2)	1 (1.2)
TDF/3TC (FTC)/NVP	15 (17.4)	16 (18.6)
TDF/3TC (FTC)/EFV	5 (5.8)	8 (9.3)
Other	0 (0.0)	1 (1.2)
Current ART regimen		
AZT/3TC/NVP	45 (52.3)	44 (51.2)
AZT/3TC/EFV	12 (14.0)	8 (9.3)
TDF/3TC (FTC)/NVP	19 (22.1)	26 (30.2)
TDF/3TC (FTC)/EFV	7 (8.1)	8 (9.3)
Any PI	3 (3.5)	0 (0.0)
Any ART switch prior to enrollment		
Yes	35 (40.7)	25 (29.1)
No	51 (59.3)	61 (70.9)
Last missed ARVs		
Last week	0 (0.0)	2 (2.3)
1–4 wk ago	5 (5.8)	2 (2.3)
1–3 mo ago	2 (2.3)	2 (2.3)
>3 mo ago	4 (4.6)	4 (4.6)
Never	75 (87.2)	76 (88.4)
Missed CPT in last month		
Yes	29 (33.7)	19 (22.1)
No	57 (66.3)	67 (77.9)
Sleeps under a mosquito net		
Yes	43 (50.0)	41 (47.7)
No	43 (50.0)	45 (52.3)

Data are presented as No. (%) unless otherwise indicated.

Abbreviations: 3TC, lamivudine; ART, antiretroviral therapy; ARV, antiretroviral; AZT, azidothymidine; BMI, body mass index; CPT, cotrimoxazole preventive therapy; d4T, stavudine; EFV, efavirenz; FTC, emtricitabine; HIV, human immunodeficiency virus; IQR, interquartile range; NVP, nevirapine; PI, protease inhibitor; SD, standard deviation; TDF, tenofovir disoproxil fumarate; WHO, World Health Organization.

### Plasma LPS Levels After CPT Discontinuation

**Figure 1.**
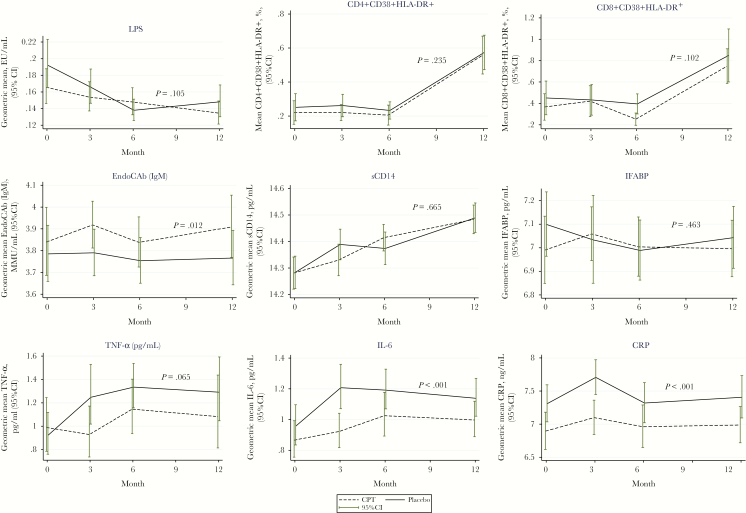
Trajectories of microbial translocation (lipopolysaccharide), T-cell immune activation, intestinal barrier damage, and inflammation markers from baseline to 3, 6, and 12 months. The plotted mean levels of markers were compared using geometric means; the *P* value in each plot refers to the linear regression model *P* value controlling for the baseline value of each marker. The dashed line shows the cotrimoxazole preventive therapy arm, complete black is the placebo arm, and the bars show the 95% confidence interval at each time point. Abbreviations: CI, confidence interval; CPT, cotrimoxazole preventive therapy; CRP, C-reactive protein; EndoCAb, anti-endotoxin core antibody; IFABP, intestinal fatty acid binding protein; IgM, immunoglobulin M; IL-6, interleukin 6; LPS, lipopolysaccharide; sCD14, soluble CD14; TNF-α, tumor necrosis factor α.

**Figure 2.**
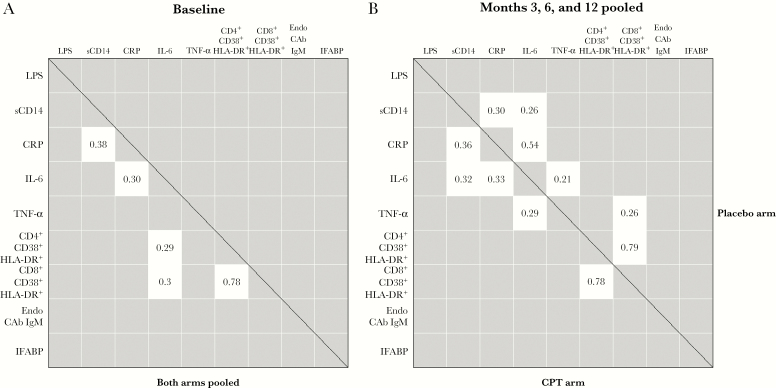
Relationships between T-cell activation, microbial translocation, and inflammation markers. Pairwise relationships were evaluated by Spearman rank correlation, with Bonferroni correction for multiple comparisons. Only ρ coefficients with a *P* value <.05 are shown. *A*, Relationships at baseline, both arms pooled. *B*, Relationships after intervention and months 3, 6, and 12 pooled, showing coefficients for the cotrimoxazole preventive therapy arm below the diagonal and placebo arm above. Abbreviations: CPT, cotrimoxazole preventive therapy; CRP, C-reactive protein; EndoCAb, anti-endotoxin core antibody; IFABP, intestinal fatty acid binding protein; IgM, immunoglobulin M; IL-6, interleukin 6; LPS, lipopolysaccharide; sCD14, soluble CD14; TNF-α, tumor necrosis factor α.

Overall, there was no significant difference in plasma LPS levels between the 2 arms (Tables 2 and 3). We observed a modest decline in LPS levels from baseline to month 12 in both arms (Figure 1). Furthermore, stopping CPT increased LPS by a mean of approximately 0.03 EU/mL and 0.07 EU/mL in the model fitted without time interaction and the model with time interaction, respectively (Table 3), though this was not statistically significant. LPS did not significantly correlate with any other markers (Figure 2). The plasma inflammatory markers IL-6, CRP, and TNF-α increased after CPT was stopped.

**Table 2.** Mean Levels of Markers at Baseline and 3, 6, and 12 Months by Treatment Arm

	Baseline (Month 0)			Month 3			Month 6			Month 12		
Marker	CPT	Placebo	*P* Value	CPT	Placebo	*P* Value	CPT	Placebo	*P* Value	CPT	Placebo	*P* Value
LPS, EU/mL	1.22^a^	0.28	.087	0.20	0.22	.397	1.21	1.16	.232	1.17	1.21	.261
TNF-α, pg/mL	2.18	2.10	.792	2.20	2.77	.088	2.97	3.56	.219	2.83	3.56	.119
sCD14, pg/mL	1 595 982	1 599 178	.850	1 672 784	1 776 223	.168	1 830 318	1 758 550	.310	1 953 240	1 966 961	.876
IL-6, pg/mL	2.74	3.04	.205	2.61	3.96	**.001**	2.71	3.26	.082	2.88	3.45	**.045**
EndoCAb IgM	49.40	46.06	.455	51.94	45.60	.103	47.94	43.82	.312	52.46	45.15	.109
IFABP, pg/mL	1118.79	1236.45	.268	1225.37	1230.28	.918	1155.17	1151.71	0.977	1118.79	1176.15	.567
CRP, ng/mL	1088.98	1649.12	**.036**	1327.43	2418.74	**.001**	1198.71	1694.26	0.108	1176.15	1872.44	**.023**
CD4^+^ CD38^+^ HLA-DR^+^, %^b^	0.22	0.25	.559	0.22	0.26	.330	0.21	0.23	0.568	0.56	0.57	.836
CD8^+^ CD38^+^ HLA-DR^+^, %^b^	0.37	0.45	.399	0.42	0.43	.929	0.25	0.38	**0.023**	0.75	0.85	.508

P values of the *t* test comparing CPT to placebo arm for each log-transformed marker at each month of follow-up. Values in bold are statistically significant.

Abbreviations: CPT, cotrimoxazole preventive therapy; CRP, C-reactive protein; EndoCAb, anti-endotoxin core antibody; IFABP, intestinal fatty acid binding protein; IgM, immunoglobulin M; IL-6, interleukin 6; LPS, lipopolysaccharide; sCD14, soluble CD14; TNF-α, tumor necrosis factor alpha.

^a^Mean (reverse log transformed).

^b^Marker not transformed on logarithm scale and indicates activation levels.

**Table 3.** Effect of Discontinuing Cotrimoxazole Preventive Therapy Analyzed by Fitting a Linear Mixed Model Without and Without Time Interaction

	LPS, EU/mL		TNF-α, pg/mL		sCD14, pg/mL		IL-6, pg/mL		EndoCAb IgM, MMU/mL		IFABP, pg/mL		CRP, ng/mL		CD4^+^ CD38^+^ HLA-DR^+^, %		CD8^+^ CD38^+^ HLA-DR^+^, %	
Model	Coef	*P* Value	Coef	*P* Value	Coef	*P* Value	Coef	*P* Value	Coef	*P* Value	Coef	*P* Value	Coef	*P* Value	Coef	*P* Value	Coef	*P* Value
Without time interaction																		
Stopping CPT	0.03	.082	0.66	**.045**	5405	.924	0.70	**.006**	–7.31	.100	42.67	.629	860.74	**< .001**	0.03	.331	0.08	.189
With time interaction																		
Stopping CPT at M0	0.07	.060	0.04	.945	–15 626	.825	0.40	.342	–6.86	.193	49.44	.673	567.32	.070	0.03	.572	0.08	.448
Stopping CPT at M3	–0.04	.410	1.16	.161	116 482	.087	0.94	.092	0.09	.984	40.93	.738	710.88	**.044**	0.01	.885	–0.06	.649
Stopping CPT at M6	–0.08	.081	0.46	.580	–46 717	.495	–0.05	.936	2.70	.561	–97.13	.431	–69.35	.845	–0.01	.982	0.05	.700
Stopping CPT at M12	–0.01	.791	0.86	.307	10 737	.876	0.26	.649	–4.61	.321	25.60	.835	519.28	.144	–0.02	.835	0.02	.901

Linear mixed models fitted with random terms for subject and random slopes over time. Values in bold are statistically significant.

Abbreviations: Coef, coefficient representing average slope over time between subjects who continue and those who stop cotrimoxazole preventive therapy; CPT, cotrimoxazole preventive therapy; CRP, C-reactive protein; EndoCAb, anti-endotoxin core antibody; IFABP, intestinal fatty acid binding protein; IgM, immunoglobulin M; IL-6, interleukin 6; LPS, lipopolysaccharide; M0, month 0; M3, month 3; M6, month 6; M12, month 12; sCD14, soluble CD14; TNF-α, tumor necrosis factor alpha.

At baseline, the means (reverse log transformed) of all soluble markers were similar in the 2 arms except CRP, which showed a statistically significant difference in the mean between the arms. We observed a statistically significant difference in the mean level of IL-6 and CRP (*P* = .001 for both) at 3 months between the study arms. This was lost at 6 months and regained at 12 months for CRP and IL-6 (*P* = .045 and *P* = .023, respectively; Table 2). Further analysis with linear mixed models showed that TNF-α, IL-6, and CRP levels significantly increased over time in the placebo arm (*P* = .045, *P* = .006, and *P* <.001, respectively) (Table 3). No significant differences between arms were observed for sCD14, IFABP, and CD4 activation markers. The frequency of activated CD8 T cells was higher (*P* = .023) in the placebo arm at 6 months (Table 3). The difference in the means between the 2 arms showed a similar pattern for TNF-α, IL-6, and CRP level from baseline to 12 months with significant rise of IL-6 and CRP in the placebo arm (*P* < .001 and *P <* .001, respectively; Figure 1). There was a trend toward a reduction of IgM endotoxin core antibody in the placebo arm compared to the CPT arm (*P* = .01; Figure 1). The largest difference in CRP, IL-6, and TNF-α levels between the CPT and placebo arms was observed at 3 months (Figure 1).

We further evaluated pairwise relationships between all markers by Spearman rank correlation. At baseline, a strong positive monotonic correlation was observed between activated CD4 and activated CD8 cell frequencies (*r* = 0.78) and weak positive correlations between CRP and sCD14 (*r* = 0.38), CRP and IL-6 (*r* = 0.30), and IL-6 and activated CD4 and activated CD8 cell frequencies (*r* = 0.30 for both) (Figure 2A). After CPT discontinuation, the 2 arms had similar positive monotonic correlations of activated CD4 and CD8 T-cell frequencies (*r* = 0.79 for placebo and *r* = 0.78 for CPT), CRP and sCD14 (*r* = 0.30 for placebo and *r* = 0.36 for CPT), CRP and IL-6 (*r* = 0.54 for placebo and *r* = 0.33 for CPT), TNF-α and IL-6 (*r* = 0.20 for placebo and *r* = 0.30 for CPT), and a weak correlation of sCD14 and IL-6 (*r* = 0.26) and activated CD8 T-cell frequencies with TNF-α (*r* = 0.26), seen in participants in the placebo arm (Figure 2B).

## DISCUSSION

This was a randomized double-blind controlled trial to test the effect of discontinuing CPT on microbial translocation and immune activation among adult Ugandans infected with HIV, clinically stable and on ART. We tested the hypothesis that CPT reduces microbial translocation from the gastrointestinal tract and thus we expected an increase in plasma LPS, inflammatory cytokines, and immune activation among HIV-infected individuals on ART when CPT was discontinued. We observed no difference in LPS between the arms from baseline to 12 months (primary outcome) and no significant correlation of LPS with any inflammatory marker. Markers for inflammation (IL-6 and CRP) and CD8 T-cell activation markers increased after CPT was stopped.

We observed increase in inflammation markers as early as 3 months after discontinuing CPT. Our finding of similar levels of plasma LPS in the CPT as in the placebo arm suggests that microbial translocation is similar in both arms, leaving the question of what might be causing the increase of inflammatory markers in the placebo arm. A direct anti-inflammatory effect of cotrimoxazole in vitro, suppressing the TNF-α and IL-6 response to LPS or zymosan in whole blood or the release of IL-8 by Caco-2 epithelial cell lines treated with IL-1β, was shown recently by Bourke et al [18]. Increased plasma IL-6, CRP, and TNF-α, but not sCD14, were observed after CPT discontinuation [18]. Alternatively, while microbial resistance to cotrimoxazole is widespread, cotrimoxazole might be altering the composition of the intestinal microbiome. In the Anti-Retroviral Research for Watoto (ARROW) study, suppression of abundance and function of gut-resident streptococci (not LPS producers) were described in Zimbabwean HIV-infected children continuing CPT, while global effects on gut microbiome were not observed and LPS-producing Enterobacteriaceae were not affected or even increased [18]. Fecal myeloperoxidase, a marker of intestinal inflammation, was lower in children continuing CPT [18]. Thus, studies in children and adults show anti-inflammatory effects of CPT not involving changes in plasma endotoxin. These mechanisms warrant further investigation.

Plasma sCD14, which binds LPS and has been shown to have a modulatory effect on the immune system [11, 19, 20], was not different between study arms. We observed a weak positive correlation of IL-6, CRP, and sCD14 in both arms, suggesting an involvement of sCD14 in the overall inflammatory response.

We observed a moderate effect of CPT on CD8 T-cell activation at 6 months. In both study arms, a rise in the activation markers CD4 and CD8 (T-cell activation) and sCD14 (monocyte activation) levels between 6 months and 12 months was observed. This could indicate loss of adherence to ART in both arms and to CPT in the CPT continuation arm or a general effect of disease progression. We did not assess systemic drug concentrations of both ART and CPT in this study. While microbial translocation was shown to increase immune activation in cross-sectional studies in HIV-infected patients from the United States [11], studies in African populations did not show such association [21, 22].

Endotoxin core antibodies are antibodies to LPS. We expected higher levels of LPS in the placebo arm, and consequently changes in EndoCAb IgM. Indeed, we observed a trend, sustained throughout the study, toward reduced IgM in the placebo arm, possibly showing increased consumption of IgM antibodies. Decrease in endotoxin core antibodies has been associated with increased endotoxin levels and increased inflammation [23].

We found no change in IFABP levels. Two other studies have shown no change of IFABP levels following CPT and ART medication among HIV-infected individuals [24, 25]. Enterocyte damage happens early in HIV infection and has not been shown to be restored even with ART [25], which may be why we observed a generally high level of IFABP in all study participants.

The strengths of our study consist of its randomized, double-blind, placebo-controlled design; the extended 1-year follow-up period with 4 standardized time points; and the broad battery of markers addressing the response to CPT discontinuation. The study has some limitations: We did not assess type I interferons stimulated by LPS via the MyD88 independent pathway. This could have given information on modulating effects in endotoxin tolerance. In addition, LAL-based assays quantify only unbound or free LPS, yet the LPS bound to lipoproteins is also biologically active and if not quantified, there is a possibility of masking the total LPS effect [26, 27]. The LAL-based assay has been shown to equally detect both the strongly endotoxic LPS and low or nonendotoxic forms of LPS [28]. We only used LPS as a marker of microbial translocation; however, analyzing blood 16S rDNA might have provided an alternative quantitative and qualitative measure of bacterial translocation. We did not examine the intestinal microbiome itself.

Increased levels of inflammatory markers can also be seen in HIV-uninfected individuals from Africa [15], and chronic inflammation has been associated with cardiovascular disease and mortality among HIV-infected adults and children on ART [29–31]. Plasma levels of the inflammatory markers IL-6 and CRP are independently associated with mortality in HIV-infected and uninfected adults [32–35] and in HIV-infected Ugandan children [30]. Although we could not confirm that CPT discontinuation was associated with microbial translocation as reflected by plasma LPS, the mechanism by which CPT may reduce systemic inflammation in the presence of intestinal barrier damage might involve modulatory mechanisms of the MyD88 independent pathway and needs further investigation.

Our findings suggest that among HIV-infected individuals stabilized on ART and CPT, CPT discontinuation does not increase microbial translocation from the gut and suggest that a beneficial effect of CPT in lowering inflammatory markers and a moderate effect on T-cell activation. These results are important for the clinical management of HIV disease and should be considered when weighing the pros and cons of CPT in resource-restricted countries.

## Supplementary Data

Supplementary materials are available at *The Journal of Infectious Diseases* online. Consisting of data provided by the authors to benefit the reader, the posted materials are not copyedited and are the sole responsibility of the authors, so questions or comments should be addressed to the corresponding author.

jiz494_suppl_Supplementary_figures

jiz494_suppl_Supplementary_table

## Notes

***Acknowledgments.*** The authors thank all COSTOP study participants at the Masaka site; Matt Price, for mentorship at the International Traineeships in AIDS Prevention Studies (ITAPS); and Pat Fast for reading and giving important discussions of the results. Members of the Medical Research Council/Uganda Virus Research Institute and the London School of Hygiene and Tropical Medicine Uganda Research Unit are listed in the Appendix.

***Author contributions.*** Conceptualization: P. P., H. G., P. K., P.M. Funding acquisition: P. P., H. G., P. K., P.M. Experiment design: P. P., J. L., J. K.-L., P. K. Performed the assays: J. K.-L., B. G., K. M. Supervision: P. P., H. G., P. K., P.M. Project administration: Z. A., J. K.-L., B. G., P.M. Formal analysis: A. A., J. L., P. P., J. K.-L.

Writing of the original draft: J. K.-L., Z. A., A. A., P. P. Manuscript review and editing: J. K.-L., Z. A., A. A., J. L., B. G., K. M., P. K., H. G., P.M., P. P. All authors read, commented on, and approved the final version of the article.

***Financial support.*** The COSTOP Immunology substudy was supported by the Medical Research Council (MRC) (award number MC_UP_1204/4, part of Quinquennial Review funding) to P. P. The COSTOP trial (grant number G0902150) was jointly funded by the UK MRC and the UK Department for International Development (DFID) under the MRC/DFID Concordat agreement and is also part of the European and Developing Countries Clinical Trials Partnership program supported by the European Union (to H. G.). J. K.-L. received protected time to draft the manuscript while on a Quantitative Biosciences Institute–University of California, San Francisco (UCSF) Scholarship for Women From Developing Nations in Biosciences to work with the Krogan laboratory. The authors acknowledge support from the UCSF ITAPS and the US National Institute of Mental Health (grant number R25MH064712) for the help they provided in drafting this manuscript.

***Potential conflicts of interest.*** The authors: No reported conflicts of interest. All authors have submitted the ICMJE Form for Disclosure of Potential Conflicts of Interest.

Presented in part: Medical Research Council/Uganda Virus Research Institute and London School of Hygiene and Tropical Medicine Uganda Research Unit Science and Advisory Meeting, Entebbe, Uganda, 9–10 May 2018.

## APPENDIX

*Medical Research Council/Uganda Virus Research Institute and the London School of Hygiene and Tropical Medicine Uganda Research Unit.* J. K. Lugemwa, P. Pala, Z. Anywaine, J. Levin, A. Abaasa, P. Kaleebu, A. Kamali, A. Mugisha, A. Nakimbugwe, A. Namara, A. Namirembe, A. Tarekwa, A. Walungama, A. N. Bukenya, A. R. Ajok, B. Gombe, B. Kalina, B. Kikaire, B. Kiyemba, B. Masiira, B. Mushabe, B. N. Kalanzi, B. Ssebunya, C. Nalubowa, C. Nampewo, C. N. Musoke, D. Katende, D. Nakitto, D. Nambuba, D. Sserunjogi, D. Wangi, E. Aling, E. Bwanika, E. Mulali, E. Zalwango, F. Kabajuma, F. Nume, F. Wamalugu, G. Bahizi, G. Barigye, G. Lubega, G. Nabaggala, G. Nabulime, G. Nassuna, G. Opio, G. Segutunga, G. Ssemwanga, H. Ayebazibwe, I. Kaddu, I. Kamulegeya, I. Namakoola, I. Ssonko, J. Bukenya, J. Bwandinga, J. Kitonsa, J. Lutaakome, J. Miiro, J. Nabakooza, J. Nabunnya, J. Namayanja, J. Namujja, J. Namutebi, J. Nsiimire, J. Okello, J. Oweka, J. Seeley, J. Were, J. K. Apuuli, K. Kugonza, L. Generous, L. Kato, L. Namayirira, L. Matama, M. Akello, M. Kwizera, M. Mbonye, M. Nakazibwe, M. Nakibuule, M. Nalaaki, M. Nalwadda, M. Namyalo, M. K. Lutwama, N. Erima, N. Kyakuwa, O. Nantume, P. Balungi, P. Hughes, P. Taire, R. Abuine, R. Kasirye, R. Nalugya, R. Namakolo, R. Nanyunja, R. Ssebukyu, S. Kaddu, S. Kizito, S. Nabalayo, S. Nassimbwa, S. Ngom-Wegi, S. Owilla, S. Tino, T. Ssempagala, T. Vudriko, V. Basajja, V. Nannono, W. Nakahima, W. Nalukenge, W. Ochola, W. Senyonga, Y. Nikweri, Z. Kamushaaga, P. Munderi.

*Medical Research Council Clinical Trials Unit, University College London, UK*. A. Nunn.

*London School of Hygiene and Tropical Medicine, UK*. H. Grosskurth.

*Partner institutions.* The AIDS Support Organisation; Uganda Cares ART clinic, Masaka; Masaka Regional Referral Hospital; Kitovu Mobile ART Clinic, Masaka; Entebbe District Hospital, Entebbe; Katabi Military Hospital, Entebbe; and Kisubi Hospital, Entebbe, Uganda.

*Trial Steering Committee.* Professor Edward Katongole Mbidde (Chair); Professor Jonathan Levin (Trial Statistician); Dr Andrew Kambugu; Dr Stephen Watiti; Dr Anatoli Kamali; Dr Morven Roberts (observer).
