# Supplementary material for: Effect of Stopping Cotrimoxazole Preventive Therapy on Microbial Translocation and Inflammatory Markers Among Human Immunodeficiency Virus–Infected Ugandan Adults on Antiretroviral Therapy: The COSTOP Trial Immunology Substudy
Source: J Infect Dis. 2019 Oct 23;222(3):381–90. doi: 10.1093/infdis/jiz494 (PMC7336573; doi:10.1093/infdis/jiz494)
Supplement: jiz494_suppl_Supplementary_figures [file jiz494_suppl_supplementary_figures.docx]

**Supplementary figure 1**

Randomized (n=172)

Stopping CPT (Placebo) (n=86)

Continuing CPT (n=86)

M0

Seen (n=83)

Seen (n=84)

Seen (n=83)

Seen (n=82)

M6

Seen (n=82)

M3

M12

Missed (n=3)

- Lost (n=3)

Missed (n=2)

- Lost (n=1)
- Not interested (1)

Missed (n=1)

- Lost (n=1)

Missed (n=2)

- Lost (n=2)

Screened (n=221)

Not eligible (n=49)

- Low CD4 (n=18)
- Defaulted (n=6)
- Grade 3 or 4 Neutropenia (n=6)
- Off CPT (n=5)
- Other (n=14)

Seen (n=80)

Missed (n=2)

- Lost (n=2)

**Supplementary figure 2**

**
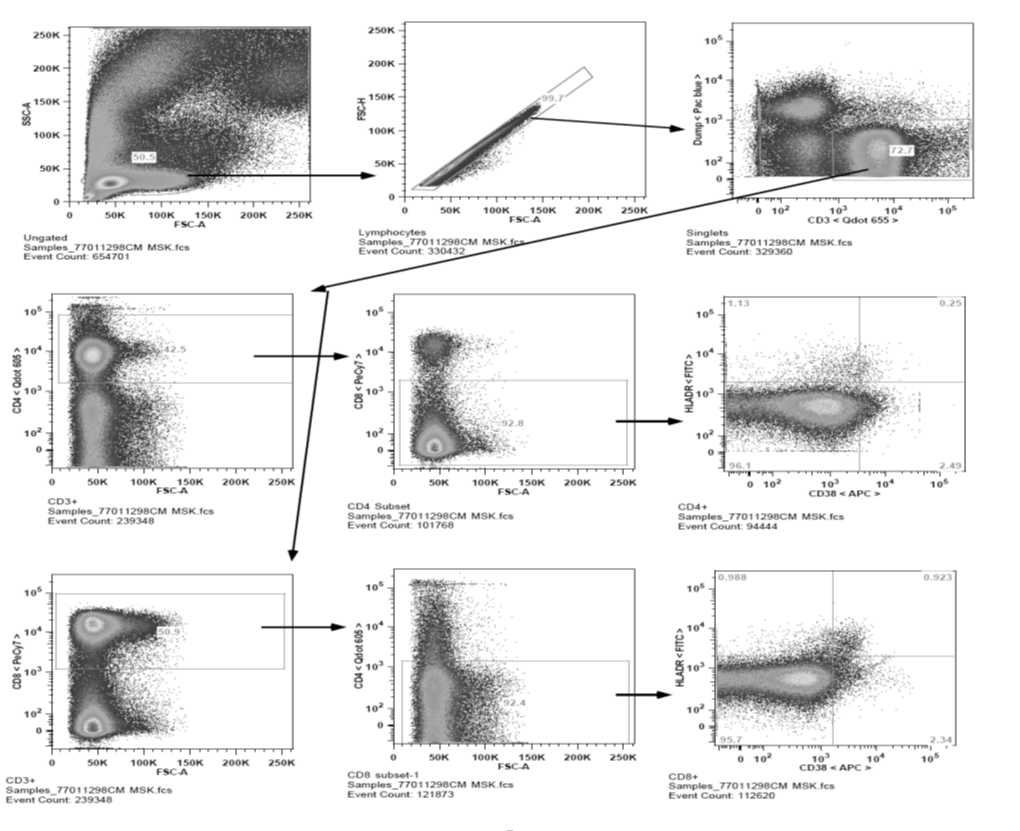
**
