# Supplementary material for: Effect of Stopping Cotrimoxazole Preventive Therapy on Microbial Translocation and Inflammatory Markers Among Human Immunodeficiency Virus–Infected Ugandan Adults on Antiretroviral Therapy: The COSTOP Trial Immunology Substudy
Source: J Infect Dis. 2019 Oct 23;222(3):381–90. doi: 10.1093/infdis/jiz494 (PMC7336573; doi:10.1093/infdis/jiz494)
Supplement: jiz494_suppl_Supplementary_table [file jiz494_suppl_supplementary_table.docx]

**Supplementary Table 1: Number of samples tested for each marker and time point.**

| **Time point**  **(Month)** | **LPS** | | **TNF-α** | | **sCD14** | | **IL-6** | | **EndoCAb**  **(IgM)** | | **IFABP** | | **CRP** | | **CD4+**  **CD38+**  **HLA-DR+*** | | **CD8+**  **CD38+**  **HLA-DR+*** | |
| --- | --- | --- | --- | --- | --- | --- | --- | --- | --- | --- | --- | --- | --- | --- | --- | --- | --- | --- |
|  | C | P | C | P | C | P | C | P | C | P | C | P | C | P | C | P | C | P |
| 0 | 83 | 81 | 81 | 79 | 79 | 78 | 81 | 79 | 81 | 78 | 78 | 72 | 81 | 79 | 82 | 79 | 82 | 79 |
| 3 | 83 | 83 | 81 | 83 | 79 | 81 | 80 | 82 | 80 | 82 | 81 | 81 | 80 | 82 | 82 | 80 | 82 | 80 |
| 6 | 78 | 79 | 77 | 79 | 78 | 79 | 78 | 79 | 78 | 79 | 78 | 78 | 78 | 79 | 78 | 76 | 78 | 76 |
| 12 | 77 | 82 | 75 | 80 | 75 | 80 | 75 | 80 | 77 | 81 | 77 | 80 | 77 | 81 | 76 | 81 | 76 | 81 |

C-Cotrimoxazole preventive therapy (CPT) arm, P-Placebo arm , LPS – Lipopolysaccharide, TNF-α - tumour necrosis factor-α, sCD14 – soluble CD14, IL-6 – Interleukin 6, EndoCAb – anti endotoxin core antibody (IgM), IFABP- Intestinal fatty acid binding protein, CRP – C-reactive protein, *CD4 and *CD8 T-cell activation markers
